# Difficult bile-duct stones in surgical anatomy managed with entero-endoscopic retrograde cholangiopancreatography and a new slim cholangioscope

**DOI:** 10.1055/a-2729-2179

**Published:** 2025-11-10

**Authors:** Marco Spadaccini, Maria Terrin, Alessandro De Marco, Giacomo Marcozzi, Matteo Colombo, Alessandro Fugazza, Alessandro Repici

**Affiliations:** 1 437807 Department of Biomedical Sciences, Humanitas University, Pieve Emanuele, Milan, Italy; 2 551905 Endoscopy Unit, IRCCS Humanitas Research Hospital, Rozzano, Milan, Italy; 3 18505 Endoscopy Unit, Humanitas Gavazzeni, Bergamo, Italy

The management of difficult biliary stones in patients with surgically altered anatomy poses a significant challenge and requires a tailored approach. Endoscopic management is preferred over percutaneous or surgical options due to lower complication rates 1 . The main difficulty lies in accessing the biliary tree. Options include creating an endoscopic ultrasound -guided gastro- or entero-enteric anastomosis 2 or performing an entero-endoscopic retrograde cholangiopancreatography (ERCP), which often limits device compatibility 3 .

A 75-year-old woman with a history of oncologic pancreatoduodenectomy presented with recurrent cholangitis and biliary lithiasis identified using magnetic resonance cholangiopancreatography. We performed an underwater cap-assisted entero-ERCP using a pediatric therapeutic colonoscope 4 . Under fluoroscopic guidance, we advanced through the afferent limb and visualized the bilioenteric anastomosis ( Fig. 1 ).

**Fig. 1:**
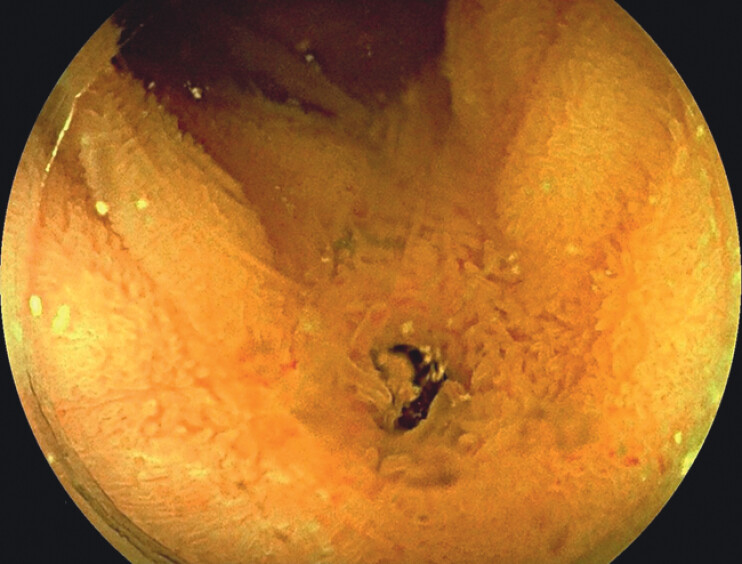
Endoscopic underwater view of the entero-biliary anastomosis using a forward-viewing therapeutic ultra-slim colonoscope (Fujifilm ELUXEO 700 Series, model EC-740T/L, with a distal end diameter of 9.8 mm and a working channel of 3.2 mm) with distal attachment; the anastomosis appears stenotic.

Cannulation of the left intrahepatic duct confirmed upstream dilation, large stones in both intrahepatic ducts, and a stenosis of the anastomosis just distal to the bifurcation ( Fig. 2 , Video 1 ).

**Fig. 2:**
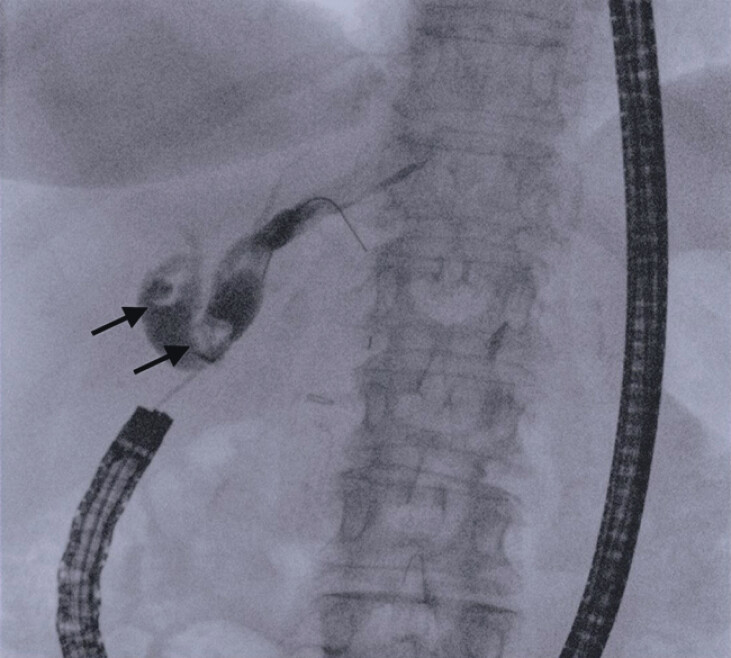
Fluoroscopic view of the biliary tree, which appears dilated with stenotic anastomosis and large (15 mm) stones in both intrahepatic ducts (black arrows).

Management of complex lithiasis in a patient with altered anatomy, combining underwater cap-assisted entero-ERCP and electrohydraulic lithotripsy using a new slim cholangioscope (2.95 mm in diameter) compatible with small-caliber working channels (3.2 mm). ERCP, endoscopic retrograde cholangiopancreatography.Video 1

We performed pneumatic dilation of the anastomosis up to 6 mm ( Fig. 3 ).

**Fig. 3:**
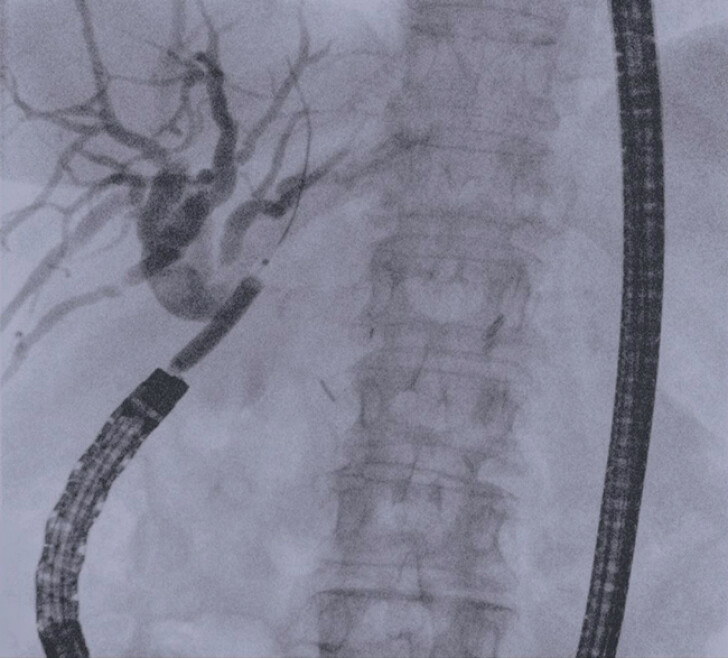
Fluoroscopic view of the inflated dilation balloon (Boston Scientific, Hurricane RX, Biliary Balloon Dilatation Catheter, 4 cm length, 6 mm diameter) passed over the left-sided guidewire through the stenotic entero-biliary anastomosis.

Given the stone size (~15 mm) and the serrated stricture, we opted for cholangioscopy-assisted electrohydraulic lithotripsy. To overcome compatibility issues with the non-operative working channel (<3.8 mm), we used a new slim Scivita cholangioscope (Boston Scientific), featuring a 2.95 mm outer diameter compatible with the 3.2 mm working channel of our pediatric scope. We accessed the left system over the wire and navigated off-wire into the right system, confirming bilateral stones. Lithotripsy was successfully performed ( Fig. 4 ), and fragments were removed using a 9–12 mm retrieval balloon.

**Fig. 4:**
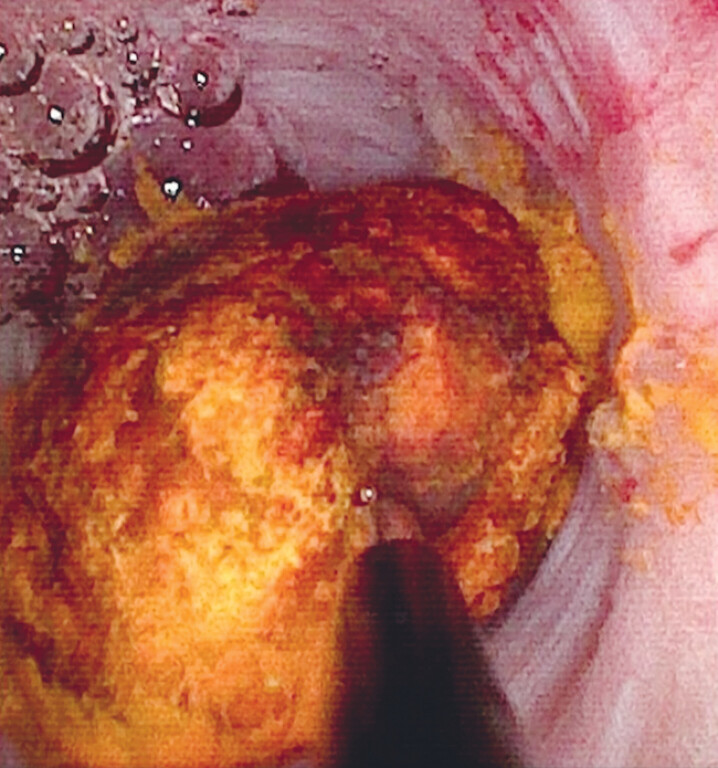
Cholangioscopic view of electrohydraulic lithotripsy of one of the stones using a Scivita single-use video cholangioscope with an outer diameter of 2.95 mm and a working channel of 1.2 mm and a biliary EHL probe (1.9 Fr diameter) with an Autolith touch EHL generator (all equipment by Boston Scientific).

Complete stone clearance was confirmed fluoroscopically and endoscopically. Two double-pigtail stents were finally placed across the anastomosis to maintain patency ( Fig. 5 ), and a follow-up endoscopy was scheduled to implement a multistenting strategy for the anastomotic stricture.

**Fig. 5:**
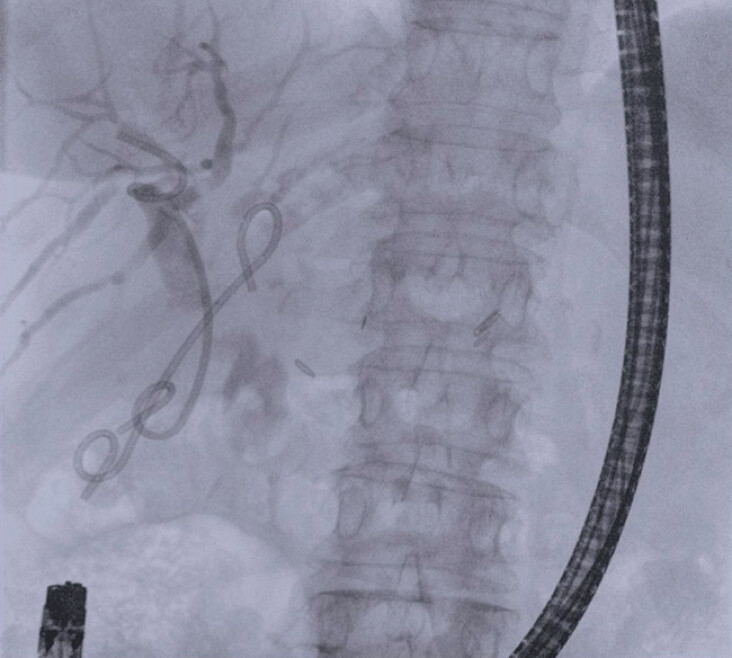
Fluoroscopic view of the two plastic double pigtail stents (7 Fr in diameter and 5 cm in length) placed across the anastomosis.

Endoscopy_UCTN_Code_TTT_1AR_2AH

## Notes

Endoscopy E-Videos https://eref.thieme.de/e-videos
*E-Videos* is an open access online section of the journal *Endoscopy* , reporting on interesting cases and new techniques in gastroenterological endoscopy. All papers include a high-quality video and are published with a Creative Commons CC-BY license. Endoscopy E-Videos qualify for HINARI discounts and waivers and eligibility is automatically checked during the submission process. We grant 100% waivers to articles whose corresponding authors are based in Group A countries and 50% waivers to those who are based in Group B countries as classified by Research4Life (see: https://www.research4life.org/access/eligibility/ ). This section has its own submission website at https://mc.manuscriptcentral.com/e-videos .
